# Stepping Up Summer Fun: the Cancer Research – Scholarship and Training Experience in Population Sciences (C-STEPS) Program

**DOI:** 10.1007/s13187-024-02458-1

**Published:** 2024-05-31

**Authors:** Paige A. Lynch, Jennifer M. Gillette, Judith N. Sheche, Shoshana Adler Jaffe, Joseph Rodman, Kate Cartwright, Miria Kano, Shiraz I. Mishra

**Affiliations:** 1 Department of Anthropology, University of New Mexico, Albuquerque, NM 87131 USA; 2 Department of Pathology, University of New Mexico Health Sciences Center, Albuquerque, NM 87131 USA; 3 Comprehensive Cancer Center, University of New Mexico, University of New Mexico Health Sciences Center, Albuquerque, NM 87131 USA; 4 Center for Healthcare Equity in Kidney Disease, University of New Mexico Health Sciences Center, Albuquerque, NM 87131 USA; 5 School of Public Administration, University of New Mexico, Albuquerque, NM 87131 USA; 6 Department of Community and Behavioral Health, Colorado School of Public Health, University of Colorado Anschutz Medical Campus, Aurora, CO 80045 USA; 7 Departments of Pediatrics and Family and Community Medicine, University of New Mexico Health Sciences Center, Albuquerque, NM 87131 USA

## Abstract

Over the last two decades, strides in cancer prevention, earlier detection, and novel treatments have reduced overall cancer mortality; however, cancer health disparities (CHD) persist among demographically diverse and intersecting populations. The development of a culturally responsive workforce trained in interdisciplinary, team-based science is a key strategy for addressing these cancer disparities. The Cancer Research – Scholarship and Training Experience in Population Sciences (C-STEPS) program at the University of New Mexico Comprehensive Cancer Center is designed to increase and diversify the biomedical and behavioral research workforce by providing specialized and experiential curricula that highlight team-oriented cancer control and population science. Undergraduate students interested in CHD and in pursuing STEM-H (science, technology, engineering, mathematics, and health) graduate or professional degrees are eligible for the program. C-STEPS students are paired with a UNM faculty mentor, who guides the student’s 10-week summer research experience. They receive mentorship and support from three layers—faculty, near-peers (graduate students), and peers (undergraduates who have completed the C-STEPS program previously). Students generate five products, including a capstone presentation, grounded in the research they conduct with their faculty mentors. Since its founding in 2021, C-STEPS has trained three cohorts with a total of 32 students. The C-STEPS program provides a unique team-science approach with multilayer mentoring to create a sustainable pipeline for the development of students interested in STEM-H fields and CHD research. The capstone project led to 47% of students presenting their work at conferences, and two publishing their manuscripts in peer-reviewed journals. Overall, 89% of students were either “satisfied” or “very satisfied” with the program and the same percentage recommended the program to other undergraduates.

**Supplementary Information:**

The online version contains supplementary material available at 10.1007/s13187-024-02458-1.

## Introduction

For more than two decades, public health efforts in the United States (US) have focused on reducing overall cancer mortality [1, 2]. Strides in cancer prevention, earlier detection, and treatments have contributed to a reduction in cancer deaths [1]. However, cancer health disparities (CHD) persist among communities with diverse and intersecting demographic characteristics, including race/ethnicity, sexual orientation and gender identity, age, socioeconomic status, geography, and health literacy [2–6]. National organizations, including the National Institutes of Health (NIH), call for the development of a culturally responsive workforce trained in interdisciplinary, team-based science to address CHD [7, 8].

In response to the President’s Council of Advisors on Science and Technology (PCAST) report [9], the “Cancer Research – Scholarship and Training Experience in Population Sciences” (C-STEPS) program was created to address the national need for more and better prepared STEM-H (science, technology, engineering, mathematics, and health) professionals [10, 11]. C-STEPS, conducted at the University of New Mexico Comprehensive Cancer Center (UNMCCC), is responsive to PCAST recommendations including (1) the use of active learning that is learner-centered rather than instructor-centered [12]; (2) employment of experiential learning in which students engage in mentored discovery-based research; and (3) facilitation of early career and skill-building opportunities via a summer research experience [11]. Mentoring design includes faculty, near-peer, and peer mentors to provide the most support for students [12]. Evidence supports that active learning increases student engagement, academic achievement, and recollection of information [13]. Likewise, experiential learning enhances student enthusiasm and confidence for a particular field, making it less likely for them to leave [14, 15]. Importantly, summer research experiences better prepare students for and retain them in STEM-H fields, improve communication skills, encourage collaboration, and increase admissions to graduate programs [11, 16–18]. Summer research programs designed to be inclusive of students from underrepresented backgrounds support national goals to diversify the workforce [19].

In addition to the PCAST guidelines, C-STEPS focuses on cancer control and population science (CCPS). New Mexico is an ideal location for a summer undergraduate research program focused on CHD and CCPS because it is one of the nation’s five “minority-majority” states based on the 2020 census data [20] and exhibits stark differences in cancer incidence, mortality, survival, and screening patterns among its multiethnic, multicultural population. The UNMCCC’s CCPS Research Program and faculty provide expertise in cancer risk and risk prediction, cancer screening, and cancer care delivery and survivorship research. C-STEPS seeks to increase and diversify the biomedical and behavioral research workforce by providing specialized, experiential, and team-oriented CCPS curricula.

In this paper, we describe the innovative characteristics of C-STEPS and summarize the outcome measures we use to evaluate the program. The program as outlined here may serve as a resource for others interested in establishing or refining a CCPS-focused summer research experience for undergraduate students.

## C-STEPS

### Program Description

C-STEPS aims to (1) develop content knowledge of cancer through inquiry-based research seminars; (2) provide a skill-based, student-oriented, mentored research experience; and (3) provide specific opportunities to implement STEM-H career development and planning. Since its inception in 2021, C-STEPS (https://unmhealth.org/cancer/career/training-education/csteps.html) has led three cohorts of students through the program (Table 1). Of the three cohorts thus far, C-STEPS students come from 19 institutions in 14 states across the US. Each year, 10–12 undergraduate students are selected to participate in a 10-week cancer research immersion experience at the University of New Mexico (UNM) Health Sciences Center (HSC) and its Comprehensive Cancer Center (CCC) in Albuquerque, NM. The students dedicate 40 hours per week to the program and receive a stipend and tuition for a one-credit biomedical seminar. C-STEPS provides student housing, and the program reimburses students for research-related expenses.

**Table 1:** Student demographics by year

Year	Cohort size	Woman:man	Hispanic:non-Hispanic	New Mexico resident:other	Accepted^†^	Waitlisted^‡^	Not accepted^§^	Did not participate^*^	Total # of applicants	Acceptance rate
2021	10	7:3	4:6	5:5	14	4	3	4	17	82%
2022	10	8:2	3:7	5:5	15	6	2	5	18	53%
2023	12	8:4	4:8	7:5	20	10	4	8	29	69%

^†^Accepted includes all students that were offered an acceptance letter

^‡^Waitlisted includes students that were on the original waitlist and may have been offered an acceptance later

^§^Not accepted students were not offered acceptance letters

^*^Students that “did not participate” were offered acceptance letters but declined the offer

C-STEPS partners with UNM HSC’s established Undergraduate Pipeline Network (UPN; https://hsc.unm.edu/medicine/education/reo/undergraduate/upn/; Jennifer Gillette, Director) summer research program to offer a focused summer research experience based on the health sciences. C-STEPS students engage in diverse learning experiences tailored to their mentor’s research project (Table 2). Students interact with the general UPN cohort through weekly seminars and team-building activities (Table 2). Students from both programs participate in the UPN Research Symposium at the end of the summer. In addition to UPN requirements, C-STEPS students participate in weekly inquiry-based cancer-focused research seminars facilitated by UNM CCC faculty. Before their arrival, students are required to complete standard research, ethics, compliance, and safety training through the CITI program and conflicts of interest training through UNM HSC [21].

**Table 2:** Program characteristics and student-generated summer research experience products

Experiences offered (*n* = 32)		
Clinical		6
Community-based		14
Wet laboratory		10
Other		2
**C-STEPS seminars**		**UPN seminars**
1. Cancer 101		1. Introduction to Research
2. The Importance of Population Health		2. Communication Skills
3. Scientific Methods		3. Professional Development
4. Risk Factors for Cancer		4. Cultural Competency
5. Cancer Screening and Early Detection		5. Team Building
6. Engaging Communities in Research		
**C-STEPS discussions**		**C-STEPS workshops**
1. Career Exploration Panel		1. Library Resources
2. Roundtable Discussion with Cancer Researchers		2. Scientific Writing
		3. Data Visualization
**Products**		
Research Poster	Research poster presentation based on a summer research project, including a 3-minute video presentation. Presented at UPN Capstone	
Research Paper	5-page research paper based on the research poster and summer research project	
Elevator Pitch	30–60 second summary of a student’s background and experience	
Digital Story	4–5-minute video summarizing the student’s C-STEPS experience, including their elevator pitch and individual development plan (IDP) synopsis. Presented at the C-STEPS Capstone	
Individual Development Plans (IDP)	A written plan, with time-defined and measurable achievements, that best reflects short- and long-term career aspirations. Students develop their IDP by following the American Association for the Advancement of Science (AAAS) web-based career-planning tool—http://myidp.sciencecareers.org/—through guidance and coaching from their faculty and near-peer mentors	

### Recruitment and Admission

C-STEPS recruits undergraduate students from across the country interested in CHD-related fields and pursuing STEM-H graduate or professional degrees. To support the diversification of a cancer-focused workforce representative of the individuals seeking cancer prevention, treatment, or survivorship care, C-STEPS supports applicants from under-represented groups within the biomedical, clinical, behavioral, and social sciences. The minimum requirements to apply include current enrollment at a university, 3.0+ GPA, 30–100 credit hours completed, and graduation no sooner than one semester after the program concludes. Additional considerations include an educational background that is consistent with C-STEPS goals and a demonstrated interest in translational cancer research. Students apply through the online UPN application portal from October to February. UPN filters applications that meet minimum qualifications and indicate interest in cancer and/or health disparities research and forwards them to the C-STEPS selection committee, composed of the program’s faculty and staff. UPN forwards applicants to C-STEPS for review each year.

Each committee member independently completes a structured assessment of each student’s coursework, previous employment and/or research/volunteer experience, research interests, personal statements, and recommendation letter(s) using a developed rubric and standardized criteria. Once completed, the combined scores are ranked. The committee organizes the applicants into “admit,” “waitlist,” and “not accepted” categories. To be accepted or on the waitlist, the student must meet all of the minimum requirements, then those with higher scores are first to be offered acceptance letters. As students accept or decline their offers, acceptance letters to the waitlisted students go out based on scores.

### Structure of the Summer Program

#### Mentorship

C-STEPS provides students with three layers of mentorship: faculty, near-peer, and peer mentors. Most C-STEPS faculty mentors are UNMCCC CCPS Research Program faculty engaged in cancer research that aligns with student interests and can accommodate student support over the summer. The faculty mentors have a variety of specialties including internal medicine, dermatology, pediatrics, population health, and nursing. Faculty mentors provide the students with hands-on, skills-based research experiences within their laboratories/teams; reinforce concepts conveyed during the weekly C-STEPS seminars; offer experience-based educational and professional perspectives; guide them through weekly challenges; and teach critical thinking skills. C-STEPS supports faculty mentors by providing a comprehensive handbook and pre-program orientation that provides onboarding to the C-STEPS program by describing the program’s aims and design, faculty mentor and student expectations, and important dates.

Near-peer mentors are graduate students who work in the UNMCCC and serve as role models, assist in research seminars and journal clubs, and provide research and career planning mentorship. The diversity in near-peer educational backgrounds, including biostatistics and anthropology, provides a variety of perspectives for C-STEPS students. In preparation for the summer, near-peers are actively involved in preparing logistical and educational components for the students. The near-peer helps students with their UPN and C-STEPS products (Table 2). Near-peer also reviews student writing throughout the summer, providing feedback on their scientific communication skills, and contributing to overall professional development.

C-STEPS also employs undergraduate peer mentors recruited from the previous year’s cohort. Six of the ten participants from the 2022 cohort applied to be peer mentors for the 2023 cohort, demonstrating sustained student commitment to the C-STEPS mission and providing a competitive pool of applicants for the peer mentor positions. Peer mentors receive a comprehensive handbook that outlines expectations and responsibilities to prepare them for their role. They attend all UPN and C-STEPS activities to provide continual, daily support to students, including first-hand guidance based on their own C-STEPS experience. Peer mentors receive the same stipend and benefits as the students.

#### Seminars, Journal Clubs, and Team-Building Activities

C-STEPS students participate in six inquiry-based research seminars (Table 2). Each seminar consists of two 1-h sessions. The original format included 2 days of lectures with discussion throughout. Based on feedback from the first two cohorts, C-STEPS adapted the seminars for the third cohort and beyond to have the first day focus on the introduction to the topic via lecture and the second day an open-group discussion. The goal of the C-STEPS seminars is to establish a baseline understanding of and highlight innovation in cancer prevention and control, surveillance, epidemiology, population health, screening and early detection, and survivorship. In addition to the six core seminars, students participate in two panel discussions (with current public health/graduate/medical students or post-doctoral fellows at UNM and cancer researchers) and three workshops (on library resources, scientific writing, and data visualization). C-STEPS requires students to attend a weekly journal club, which provides a more informal forum for discussion and engagement of research related to corresponding weekly seminar topics. There are a total of nine journal clubs and each student rotates through a co-leadership role for the discussions, assisted by the near-peer and peer mentors. Concurrent with the C-STEPS curriculum, students participate in the UPN program syllabus, which introduces more generalizable lessons on research, ethical conduct, cultural competency, communication skills, and professional development skills.

UPN and C-STEPS host various team-building activities and events for students to develop bonds with their peers, learn about values and decision-making from people of diverse backgrounds and cultures, and dispel myths and stereotypical perceptions. The goal of these team-building activities is to provide the students with skills to excel in the realm of team science. Examples of team-building activities include attending the Cochiti Pueblo Feast Day (a celebration of Native American traditions with traditional dances, cultural activities, food, and arts and crafts), campus scavenger hunts, rock climbing, and river rafting on the Rio Grande.

#### Capstone Products and Events

There are three main types of mentored experiences accessible to students depending on their mentors and projects available at the time, clinical, community-based, and wet laboratory. Experiences categorized as “other” include projects using national datasets or scoping reviews. There are 16 C-STEPS and UPN Seminars and the C-STEPS Panels and Workshops. Five C-STEPS student-generated summer research experience products are presented at two Capstone events (Table 2). Together, the development and presentation of these products serve as formative team-based research experiences, critical early career contributions to resumes, and stepping-stones for submissions to conferences (as abstracts) and journals (as manuscripts) in collaboration with their faculty mentors and teams. For example, one student in collaboration with their faculty mentor published a manuscript in the peer-reviewed journal *Early Intervention in Psychiatry* based on data from their C-STEPS project [22].

### Program Evaluation

Pre-/post-seminar surveys measure changes in knowledge among C-STEPS students in current and emerging CCPS topics, self-efficacy in research skills, confidence to succeed in the STEM-H field, comfort with research, and career aspiration. The Capstone Event poster and virtual presentations provide an opportunity for formal feedback on students’ science communication skills. The long-term tracking of C-STEPS students’ academic trajectory will be conducted through the National Student Clearinghouse, which provides access to enrollment and degree completion information. Bi-annual “alumni surveys” in REDCap track the long-term achievements of students—asking if students chose to pursue graduate or professional schools and about other information related to the utilization of the research they conducted during the program (e.g., awards, research dissemination). At the end of each summer, the C-STEPS program team conducts interviews with the faculty mentors and focus groups with the students to evaluate areas for improvement and highlight successes.

Throughout the program, we discuss both short-term and long-term outcomes with students. Short-term outcomes include presenting their research at regional and national conferences and publishing in national journals. Currently, 47% of C-STEPS students have presented their research at conferences, and four have submitted manuscripts to peer-reviewed journals, with two accepted. Looking ahead, the program aims for long-term outcomes such as students pursuing graduate or professional schools related to STEM-H fields and building careers in cancer biology or population science. The January 2024 alumni survey showed that out of 27 respondents, 18 are enrolled in undergraduate programs, eight have graduated, and one is in medical school. When asked about their primary long-term career goals, there were 26 responses from all three cohorts. Forty-six percent wish to practice medicine, 19% to work in allied health professions, 15% in industry research, 12% in university research, 4% in public health with underprivileged communities, and 4% to pursue a master’s in public health. While many C-STEPS students are still pursuing their undergraduate degrees, they have shared feedback on the program’s impact. One student noted, “C-STEPS has made it much more efficient to integrate yourself into the research world. After leaving, it was simple to reach out to researchers.” Another student mentioned, “C-STEPS helped me tremendously to confidently seek out mentors to teach and guide my professional and academic development. It has also solidified my passion for a healthcare career, specifically treating patients of marginalized backgrounds.”

Through three cohorts, students clearly and consistently indicate they feel more prepared for graduate school after completing their C-STEPS summer research experience. This is consistent with our findings from the January 2024 alumni survey in which 89% of respondents reported being “satisfied” or “very satisfied” with the C-STEPS program. One student stated: “C-STEPS has helped me continue working in research and helped with my application/drive to pursue a medical career. C-STEPS has [created] opportunities for academic/research growth.” Another student noted: “My experience studying toxicology [with C-STEPS] has convinced me to integrate my passion for research into my long-term career plans, where before this summer I was not sure if I would want a career in the basic sciences following graduation.” Overall, student satisfaction with the C-STEPS program is high and 89% of individuals have suggested that other undergraduates should apply.

### Innovation

C-STEPS augments the NIH’s cancer-focused summer research experience program framework with several distinctive and innovative design features [18, 23, 24]. First, C-STEPS provides multilayered mentoring from faculty, near-peers, and peer mentors to facilitate learning from multiple mentors in various stages of their research careers. Second, the program models the importance of team education and team science to create a sustainable pipeline for cancer research. Such an environment replicates the real-world collaborative science to which future scholars will contribute. Third, C-STEPS prioritizes career exploration and planning by exposing students to a variety of undergraduate, graduate, and professional pathways and perspectives from the multiple layers of mentorship. In addition to NIH’s framework, the C-STEPS program has implemented many innovations to achieve our aims better and make the research experience more accessible to the students (Table 3). These innovations are based on feedback from students, mentors, peer mentors, and the C-STEPS team members throughout the past three summers.

**Table 3:** Summary of innovations implemented during the summers of 2021–2023 based on the three aims of C-STEPS and student feedback

Aims	Innovation	Implementation
1: Develop content knowledge of cancer through inquiry-based research seminars	Hybrid model for COVID	• Provided option for students, study team members, mentors, faculty seminar leaders, and others to attend and participate remotely or in-person • Secured access to Health Sciences Center laptops for students to loan on as-needed basis
	American Association for Cancer Education (AACE) engagement lounge	• Forwarded registration and webinar link to C-STEPS 2023 cohort
	Active learning workshops	• Provide 90-minute training on conducting PubMed searches and Zotero, offered by a Health Sciences Library Information Systems specialist
	National Cancer Institute (NCI) seminars	• Require students to attend NCI Rising Scholars: Cancer Research Seminar Series • Require students to listen to “Inside Cancer Careers” podcast
2: Provide a skill-based, student-oriented, mentored research experience	Responsible conduct of research (RCR) training via CITI	• Require students to participate in formal CITI RCR training in addition to their CITI Group 1 or 2 training
	Clinical and in-person experience	• Provide in-person experiences with mentors • Offer information to students from UNMH volunteers for shadowing opportunity
3: Provide specific opportunities to implement STEM-H career development and planning	Professional development opportunities	• Students produce Digital Stories that present their reflections from the summer and Individual Development Plans • Provide a Career Exploration Panel for students to meet with graduate students, medical students, and post-doctoral fellows • Provide a Cancer Researchers Panel for students to meet with faculty and clinicians • Require students to conduct at least three “informational interviews” with faculty at UNM or their home institution
	Information sessions	• Offer students support for conference preparation • Offer FAQ sessions for students via Zoom for summer program preparation
	Letter of intent (LOI) for peer mentors	• Require students to send an LOI to C-STEPS if interested in a peer mentor position
Feedback from Students	Faculty, student, and seminar speaker awards	• Provide “Most Impactful” and “Most Engaging” awards for faculty seminar speakers • Provide each student with a certificate of completion and acknowledgment of individuality
	Office hours and workspace	• Offer 40 hours/week of virtual and in-person office hours by the near-peer and peer mentors • Reserve classrooms that had requisite space for social distancing each day of the summer program for students to use to conduct their work and interact with others
	UPN application	• List C-STEPS description on the application and added epidemiology as a research interest option
	Basic living items upon arrival to UNM	• Provide kitchen supplies and cookware for students to reduce student burden

### Limitations

C-STEPS has at times encountered challenges communicating with students and mentors, resulting in an occasional lack of clarity regarding program expectations. To address this challenge, C-STEPS developed and distributed comprehensive handbooks for mentors, peer mentors, and students. To date, nearly all of the mentor-mentee pairs have been productive, collaborative, and yielded positive experiences for both parties. The mentor-mentee match is fundamental to student success. For matches that did not pair well, C-STEPS promptly and discreetly discussed the concerns with the mentor and the student. In the rare event of an irresolvable situation, C-STEPS reassigned the student to a different mentor who was available and identified as a better fit. Based on these experiences, we continue to fine-tune the process to match students and faculty mentors. For current R25 PIs, or those looking to start an R25, we recommend the following:

1. Create a system for involving and coordinating peer mentors to support the participant-peer mentor connection effectively.
2. Collaborate with mentors who can commit to weekly meetings with their mentees.
3. Partner with mentors who are actively engaged in their projects to provide more effective guidance to their mentees.

## Conclusions

C-STEPS is generating a pipeline of well-rounded translational researchers. The C-STEPS program is distinctive in its multilayered mentorship structure and its location in New Mexico. As the only summer research experience focusing on CCPS in the state of New Mexico, our program exposes students to geographically and demographically diverse environments and health disparities not experienced in other parts of the country. Furthermore, C-STEPS students have been consistently showcasing their research at national conferences, in which there have been 11 presentations thus far. Overall, the experiences of the C-STEPS program may serve as a model to establish or refine a CCPS-focused summer research experience for undergraduate students.

## Electronic Supplementary Material

Below is the link to the electronic supplementary material.

Supplementary Material 1

Supplementary Material 2
