# Supplementary material for: Stepping Up Summer Fun: the Cancer Research – Scholarship and Training Experience in Population Sciences (C-STEPS) Program
Source: J Cancer Educ. 2024 May 31;40(1):3–10. doi: 10.1007/s13187-024-02458-1 (PMC11607144; doi:10.1007/s13187-024-02458-1)
Supplement: Supplementary file 1 — Supplementary Material 1 [file 13187_2024_2458_MOESM1_ESM.docx]

**Acknowledgment:** The authors also gratefully acknowledge the project’s External Advisory Committee members (Drs. Shine Chang, Jani Ingram, and Robin Harris) for their thoughtful insights and comments on the manuscript and the contributions of Monica Asencio Pimentel, who participated in the early stages of this manuscript’s development.
