# Supplementary material for: Stepping Up Summer Fun: the Cancer Research – Scholarship and Training Experience in Population Sciences (C-STEPS) Program
Source: J Cancer Educ. 2024 May 31;40(1):3–10. doi: 10.1007/s13187-024-02458-1 (PMC11607144; doi:10.1007/s13187-024-02458-1)
Supplement: Supplementary file 2 — Supplementary Material 2 [file 13187_2024_2458_MOESM2_ESM.docx]

**Funding:** This research was supported in part by the National Cancer Institute of the National Institutes of Health grant R25CA247677 (Mishra, PI) and support from the UNM Comprehensive Cancer Center (UNMCCC). This research used services provided by the Behavioral Measurement and Population Science Shared Resource and the Biostatistics Shared Resource, facilities supported by the State of New Mexico, and the UNMCCC Support Grant (P30CA118100; Sanchez, PI).
